# Asbury Park and the Conventions

**Published:** 1895-06

**Authors:** 


### ASBURY PARK AND THE CONVENTIONS.

Upon another page the attention of members of the American Dental Association, as well as societies, is called to the rapidly approaching meeting at Asbury Park, August 6. It is confidently anticipated that this will be one of the largest gatherings this body has held in years, and we see no reason why this hope should not be realized. The place in many respects is an ideal one for a summer convention, the only drawback being its extreme eastern location.

The Local Committee has secured the large auditorium for the meetings and exhibits. This is located near the board walk and the ocean, and is one of the coolest spots in the Park. It is quite important that the various organizations throughout the country should send delegates. The meetings ordinarily are composed of permanent members, and while this may be necessary to complete the organization, the vigorous life of new blood is sadly needed. Let the various societies see to it that delegates are appointed who will be in attendance.

We are informed it is the intention of the Local Committee to issue a map of the Park and give all necessary information. It is hoped this may include the names and locations of a portion, at least, of hotels and cottages. It is quite important that rooms should be secured some time in advance, for it must be remembered that while Asbury Park is capable of absorbing immense crowds, it is always very full in August.

